# 2-Hydr­oxy-3,3-dimethyl-7-nitro-3,4-dihydro­isoquinolin-1(2*H*)-one

**DOI:** 10.1107/S1600536808013457

**Published:** 2008-05-10

**Authors:** Hassen Ben Salah, Majed Kammoun, Besma Hamdi, Luis Bohé, Mohamed Damak

**Affiliations:** a Laboratoire de Chimie des Substances Naturelles, Faculté des Sciences de Sfax, BP 1171, 3000 Sfax, Tunisia; b Laboratoire de Sciences de Matériaux et d’Environnement, Faculté des Sciences de Sfax, BP 1171, 3000 Sfax, Tunisia; c ICSN–CNRS, 1 avenue de la Terrasse, 91198 Gif sur Yvette, France

## Abstract

In the title compound, C_11_H_12_N_2_O_4_, a new hydroxamic acid which belonging to the isoquinole family, the heterocyclic ring adopts a half-chair conformation. The nitro group is essentially coplanar with the aromatic ring. Inter­molecular O—H⋯O hydrogen bonds assemble the mol­ecules around inversion centres to form pseudo-dimers.

## Related literature

For related literature, see: Bohé & Kammoun (2004 ▶); Kurzak *et al.* (1992 ▶); Porcheddu & Giacomelli (2006 ▶); Weber (1983 ▶); Miller (1989 ▶); Cremer & Pople (1975 ▶).
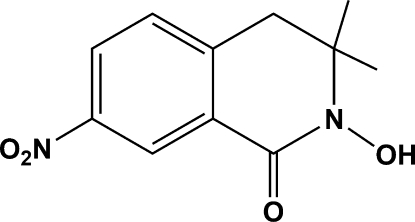


## Experimental

### 

#### Crystal data

C_11_H_12_N_2_O_4_
*M* *_r_* = 236.23
Monoclinic, 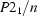

*a* = 5.8805 (9) Å
*b* = 18.605 (4) Å
*c* = 10.1588 (17) Å
β = 103.056 (12)°
*V* = 1082.7 (3) Å^3^
*Z* = 4
Mo *K*α radiation
μ = 0.11 mm^−1^
*T* = 296 K
0.60 × 0.51 × 0.22 mm

#### Data collection

Bruker SMART CCD area-detector diffractometer
Absorption correction: multi-scan (Coppens *et al.*, 1965 ▶) *T*_min_ = 0.938, *T*_max_ = 0.975
7659 measured reflections
3286 independent reflections
2051 reflections with *I* > 2σ(*I*)
*R*_int_ = 0.021

#### Refinement

*R*[*F* ^2^ > 2σ(*F* ^2^)] = 0.044
*wR*(*F* ^2^) = 0.150
*S* = 1.11
3286 reflections
157 parameters
H-atom parameters constrained
Δρ_max_ = 0.26 e Å^−3^
Δρ_min_ = −0.27 e Å^−3^

### 

Data collection: *SMART* (Bruker, 1998 ▶); cell refinement: *SAINT* (Bruker, 1998 ▶); data reduction: *SAINT*; program(s) used to solve structure: *SHELXS97* (Sheldrick, 2008 ▶); program(s) used to refine structure: *SHELXL97* (Sheldrick, 2008 ▶); molecular graphics: *ORTEPIII* (Burnett & Johnson, 1996 ▶), *ORTEP-3 for Windows* (Farrugia, 1997 ▶) and *PLATON* (Spek, 2003 ▶); software used to prepare material for publication: *WinGX* (Farrugia, 1999 ▶).

## Supplementary Material

Crystal structure: contains datablocks I, global. DOI: 10.1107/S1600536808013457/dn2332sup1.cif

Structure factors: contains datablocks I. DOI: 10.1107/S1600536808013457/dn2332Isup2.hkl

Additional supplementary materials:  crystallographic information; 3D view; checkCIF report

## Figures and Tables

**Table 1:** Hydrogen-bond geometry (Å, °)

*D*—H⋯*A*	*D*—H	H⋯*A*	*D*⋯*A*	*D*—H⋯*A*
O12—H12⋯O11^i^	0.82	1.99	2.7013 (14)	144
O12—H12⋯O11	0.82	2.20	2.6305 (15)	113

Symmetry code: (i) 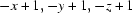.

**Fig. 1.**
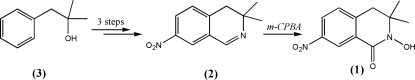
Chemical pathway of the formation of hydroxamic acid.

**Fig. 2.**
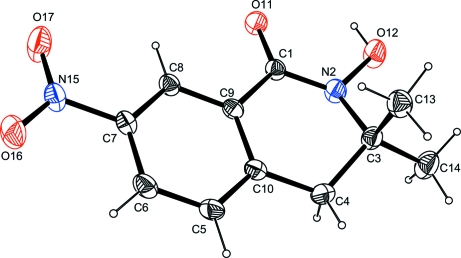
Molecular view of the title compound with the atom-labelling scheme. Ellispsoids are drawn at the 30% probability level. H atoms are represented as small spheres of arbitrary radii.

**Fig. 3.**
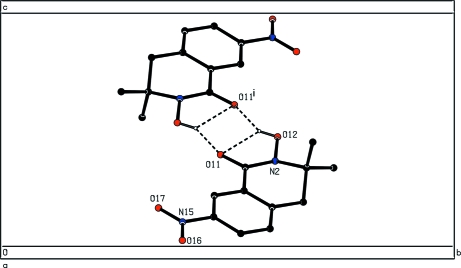
Partial packing view showing the formation of pseudo dimer through O-H···O hydrogen bonds. Hydrogen bonds are shown as dashed lines. H atoms not involved in hydrogen bondings have been omitted for clarity. [Symmetry code: (i) 1-x, 1-y, 1-z]

## supplementary crystallographic information

### Comment

Hydroxamic acids are strong metal ion chelators (Kurzak *et al.*, 1992), they posses a wide spectrum of biological activities, such as antibacterial, antifungal, anti-inflammatory, and anti-asthmatic properties, *etc*. (Weber, 1983; Miller 1989).

The growing number of published synthetic methods further points to the biological significance of hydroxamic acids (Porcheddu & Giacomelli, 2006). Among this family of hydroxamic acids is the title compound (1). We report herein its synthesis and its crystal structure determination. Synthesis of the title compound has been prepared from the corresponding dihydroisoquinoleine (2) by metachloroperbenzoic acid oxidation (Fig. 1). Imine (2) was described by Bohé and Kammoun (2004), in three steps from the commercially available tertiary alcohol (3).

In the title compound, the heterocyclic ring adopts a half-chair conformation as indicated by the puckering parameters: Q = 0.4224 (14)Å, θ = 57.87 (18)°, φ = 281.2 (2)° (Cremer & Pople, 1975). The nitro group attached on C7 is essentially coplanar with the aromatic nucleus (Fig. 2). The methyl substituent in position 3 of the heterocyclic ring is pseudo-axial, the second methyl in position 3 is pseudo-equatorial.

The conformation of (1) is stabilized by an intramolecular hydrogen bond between the hydroxyl O12—H12 group and atom O11 (Table 1).The molecules are assembled by intermolecualr O-H···O hydrogen bonds to form pseudo dimer arranged around inversion centre (Table 1, Fig. 3)

### Experimental

The title compound was prepared by reaction of imine (2) (100 mg, 0.49 mmol), and methachloroperbenzoique acid 86% (197 mg, 0.98 mmol) in dichloromethane (15 ml). The mixture was stirred at room temperature for 24 h. Then, the mixture was diluted with CH_2_Cl_2_ and washed with a solution of saturated NaHCO_3_. The organic phase was dried over sodium sulfate, filtered and concentrated under reduced pressure. The concentrate was chromatographed on silica gel, with (ether) as eluent (yield 40%). m.p.418 K. Spectroscopic analysis, 1H NMR (300 MHz; DMSO-d_6_, p.p.m): 1.26 (s, 6H, 2Me 3); 3.23 (s, 2H); 7.59 (d, J = 8.4, 1H, aromatic H); 8.33 (dd, J = 8.4, J= 2.4, 1H, aromatic H); 8.56 (d, J = 2.4, 1H aromatic H); 9.8 (s wide, 1H, OH). 13 C NMR (75 MHz; DMSO-d_6_, p.p.m): 25.24; 41.80; 60.66; 122.02; 126.87; 129.91;130.41; 144.05; 147.21; 160.08. M.S (EI, 70 ev): m/*z*: 236 (*M*+.); 221 [(M–15)+., base peak]. MS (HR): Found: 236,0844 calcd mass for C_11_H_12_N_2_O_4_: 236,0875. Recrystallizations from dichloromethane afford yellow crystals suitable for diffraction.

### Refinement

All H atoms attached to C atoms and O atom were fixed geometrically and treated as riding with C—H = 0.98 Å (methyl), 0.97 Å (methylene), 0.93Å (aromatic) and O—H = 0.82 Å with U_iso_(H) = 1.2U_eq_(C) or U_iso_(H) = 1.5U_eq_(O, C_aromatic_).

### Crystal data

**Table:**

C_11_H_12_N_2_O_4_	*F*_000_ = 496
*M**_r_* = 236.23	*D*_x_ = 1.449 Mg m^−^^3^
Monoclinic, *P*2_1_/*n*	Melting point: 418 K
Hall symbol: -P 2yn	Mo *K*α radiation λ = 0.71073 Å
*a* = 5.8805 (9) Å	Cell parameters from 2421 reflections
*b* = 18.605 (4) Å	θ = 2.5–23.2º
*c* = 10.1588 (17) Å	µ = 0.11 mm^−^^1^
β = 103.056 (12)º	*T* = 296 K
*V* = 1082.7 (3) Å^3^	Prism, colourless
*Z* = 4	0.60 × 0.51 × 0.22 mm

### Data collection

**Table:**

Bruker SMART CCD area-detector diffractometer	3286 independent reflections
Radiation source: sealed tube	2051 reflections with *I* > 2σ(*I*)
Monochromator: graphite	*R*_int_ = 0.021
*T* = 296 K	θ_max_ = 30.4º
φ and ω scans	θ_min_ = 2.2º
Absorption correction: multi-scan(Coppens *et al.*, 1965)	*h* = −8→8
*T*_min_ = 0.938, *T*_max_ = 0.975	*k* = 0→26
7659 measured reflections	*l* = 0→14

### Refinement

**Table:**

Refinement on *F*^2^	Secondary atom site location: difference Fourier map
Least-squares matrix: full	Hydrogen site location: inferred from neighbouring sites
*R*[*F*^2^ > 2σ(*F*^2^)] = 0.044	H-atom parameters constrained
*wR*(*F*^2^) = 0.151	*w* = 1/[σ^2^(*F*_o_^2^) + (0.0543*P*)^2^ + 0.0949*P*] where *P* = (*F*_o_^2^ + 2*F*_c_^2^)/3
*S* = 1.11	(Δ/σ)_max_ = 0.001
3286 reflections	Δρ_max_ = 0.26 e Å^−^^3^
157 parameters	Δρ_min_ = −0.27 e Å^−^^3^
Primary atom site location: structure-invariant direct methods	Extinction correction: none

### Special details

**Table:**

Geometry. All esds (except the esd in the dihedral angle between two l.s. planes) are estimated using the full covariance matrix. The cell esds are taken into account individually in the estimation of esds in distances, angles and torsion angles; correlations between esds in cell parameters are only used when they are defined by crystal symmetry. An approximate (isotropic) treatment of cell esds is used for estimating esds involving l.s. planes.
Refinement. Refinement of *F*^2^ against ALL reflections. The weighted *R*-factor *wR* and goodness of fit *S* are based on *F*^2^, conventional *R*-factors *R* are based on *F*, with *F* set to zero for negative *F*^2^. The threshold expression of *F*^2^ > σ(*F*^2^) is used only for calculating *R*-factors(gt) *etc*. and is not relevant to the choice of reflections for refinement. *R*-factors based on *F*^2^ are statistically about twice as large as those based on *F*, and *R*- factors based on ALL data will be even larger.

### Fractional atomic coordinates and isotropic or equivalent isotropic displacement parameters (Å^2^)

**Table:**

	*x*	*y*	*z*	*U*_iso_*/*U*_eq_	
C1	0.7652 (2)	0.53911 (7)	0.36105 (13)	0.0323 (3)	
C3	0.7990 (2)	0.67410 (7)	0.36068 (15)	0.0380 (3)	
C4	0.8660 (3)	0.66689 (8)	0.22427 (15)	0.0426 (3)	
H4A	0.7256	0.6692	0.1527	0.051*	
H4B	0.9645	0.7072	0.2127	0.051*	
C5	1.1554 (3)	0.59404 (8)	0.13045 (14)	0.0393 (3)	
H5	1.1903	0.6347	0.0857	0.047*	
C6	1.2662 (3)	0.52948 (8)	0.11699 (14)	0.0410 (3)	
H6	1.3779	0.5268	0.0653	0.049*	
C7	1.2075 (2)	0.46956 (7)	0.18163 (13)	0.0339 (3)	
C8	1.0435 (2)	0.47108 (7)	0.25982 (13)	0.0330 (3)	
H8	1.0052	0.4297	0.3015	0.040*	
C9	0.9371 (2)	0.53674 (7)	0.27418 (12)	0.0296 (2)	
C10	0.9923 (2)	0.59838 (7)	0.21048 (13)	0.0338 (3)	
C13	1.0066 (3)	0.68808 (8)	0.47591 (16)	0.0471 (3)	
H13A	0.9566	0.6890	0.5596	0.071*	
H13B	1.0752	0.7335	0.4625	0.071*	
H13C	1.1200	0.6506	0.4789	0.071*	
C14	0.6172 (3)	0.73411 (8)	0.35087 (19)	0.0538 (4)	
H14A	0.4823	0.7226	0.2813	0.081*	
H14B	0.6829	0.7787	0.3294	0.081*	
H14C	0.5727	0.7386	0.4358	0.081*	
N2	0.6880 (2)	0.60494 (6)	0.38233 (13)	0.0402 (3)	
N15	1.3238 (2)	0.40117 (7)	0.16643 (13)	0.0438 (3)	
O11	0.69294 (19)	0.48475 (5)	0.40857 (11)	0.0437 (3)	
O12	0.5448 (2)	0.61049 (6)	0.47313 (13)	0.0542 (3)	
H12	0.5129	0.5702	0.4961	0.081*	
O16	1.4723 (3)	0.40057 (7)	0.09958 (16)	0.0701 (4)	
O17	1.2691 (3)	0.34788 (6)	0.22078 (16)	0.0724 (5)	

### Atomic displacement parameters (Å^2^)

**Table:**

	*U*^11^	*U*^22^	*U*^33^	*U*^12^	*U*^13^	*U*^23^
C1	0.0312 (5)	0.0344 (6)	0.0338 (6)	−0.0011 (5)	0.0129 (4)	−0.0013 (5)
C3	0.0393 (7)	0.0316 (6)	0.0463 (8)	0.0024 (5)	0.0163 (5)	0.0012 (5)
C4	0.0514 (8)	0.0382 (7)	0.0403 (7)	0.0039 (6)	0.0146 (6)	0.0078 (5)
C5	0.0468 (8)	0.0409 (7)	0.0340 (7)	−0.0071 (5)	0.0172 (5)	0.0027 (5)
C6	0.0422 (7)	0.0506 (8)	0.0357 (7)	−0.0047 (6)	0.0203 (5)	−0.0025 (6)
C7	0.0331 (6)	0.0385 (7)	0.0323 (6)	−0.0022 (5)	0.0118 (4)	−0.0066 (5)
C8	0.0341 (6)	0.0356 (6)	0.0316 (6)	−0.0023 (4)	0.0125 (4)	−0.0028 (5)
C9	0.0251 (5)	0.0356 (6)	0.0300 (5)	−0.0015 (4)	0.0102 (4)	−0.0009 (4)
C10	0.0369 (6)	0.0378 (7)	0.0283 (6)	−0.0015 (5)	0.0104 (4)	0.0001 (5)
C13	0.0491 (8)	0.0449 (8)	0.0478 (8)	−0.0067 (6)	0.0120 (6)	−0.0062 (6)
C14	0.0549 (10)	0.0395 (8)	0.0710 (11)	0.0094 (7)	0.0224 (8)	0.0025 (7)
N2	0.0398 (6)	0.0384 (6)	0.0484 (7)	0.0012 (5)	0.0225 (5)	−0.0004 (5)
N15	0.0435 (7)	0.0449 (7)	0.0484 (7)	−0.0015 (5)	0.0216 (5)	−0.0107 (5)
O11	0.0474 (6)	0.0392 (5)	0.0530 (6)	−0.0016 (4)	0.0290 (5)	0.0036 (4)
O12	0.0577 (7)	0.0439 (6)	0.0759 (8)	0.0026 (5)	0.0466 (6)	−0.0024 (5)
O16	0.0803 (10)	0.0621 (8)	0.0872 (10)	0.0148 (7)	0.0593 (9)	0.0031 (7)
O17	0.0887 (11)	0.0377 (6)	0.1104 (12)	−0.0020 (6)	0.0633 (10)	−0.0055 (7)

### Geometric parameters (Å, °)

**Table:**

C1—O11	1.2366 (15)	C7—C8	1.3816 (17)
C1—N2	1.3407 (16)	C7—N15	1.4690 (18)
C1—C9	1.4851 (16)	C8—C9	1.3950 (17)
C3—N2	1.4818 (18)	C8—H8	0.9300
C3—C13	1.511 (2)	C9—C10	1.3905 (17)
C3—C4	1.530 (2)	C13—H13A	0.9600
C3—C14	1.533 (2)	C13—H13B	0.9600
C4—C10	1.497 (2)	C13—H13C	0.9600
C4—H4A	0.9700	C14—H14A	0.9600
C4—H4B	0.9700	C14—H14B	0.9600
C5—C6	1.388 (2)	C14—H14C	0.9600
C5—C10	1.3927 (18)	N2—O12	1.3862 (14)
C5—H5	0.9300	N15—O17	1.2130 (17)
C6—C7	1.3768 (19)	N15—O16	1.2215 (16)
C6—H6	0.9300	O12—H12	0.8200
			
O11—C1—N2	121.69 (11)	C9—C8—H8	121.1
O11—C1—C9	123.22 (11)	C10—C9—C8	121.09 (11)
N2—C1—C9	115.07 (11)	C10—C9—C1	120.91 (11)
N2—C3—C13	109.91 (12)	C8—C9—C1	118.00 (11)
N2—C3—C4	105.70 (11)	C9—C10—C5	119.18 (12)
C13—C3—C4	112.86 (12)	C9—C10—C4	119.07 (12)
N2—C3—C14	108.53 (12)	C5—C10—C4	121.68 (12)
C13—C3—C14	110.76 (13)	C3—C13—H13A	109.5
C4—C3—C14	108.88 (12)	C3—C13—H13B	109.5
C10—C4—C3	113.20 (11)	H13A—C13—H13B	109.5
C10—C4—H4A	108.9	C3—C13—H13C	109.5
C3—C4—H4A	108.9	H13A—C13—H13C	109.5
C10—C4—H4B	108.9	H13B—C13—H13C	109.5
C3—C4—H4B	108.9	C3—C14—H14A	109.5
H4A—C4—H4B	107.8	C3—C14—H14B	109.5
C6—C5—C10	120.56 (12)	H14A—C14—H14B	109.5
C6—C5—H5	119.7	C3—C14—H14C	109.5
C10—C5—H5	119.7	H14A—C14—H14C	109.5
C7—C6—C5	118.67 (12)	H14B—C14—H14C	109.5
C7—C6—H6	120.7	C1—N2—O12	117.02 (11)
C5—C6—H6	120.7	C1—N2—C3	126.35 (11)
C6—C7—C8	122.71 (12)	O12—N2—C3	112.85 (10)
C6—C7—N15	118.59 (11)	O17—N15—O16	122.81 (13)
C8—C7—N15	118.70 (12)	O17—N15—C7	118.86 (12)
C7—C8—C9	117.77 (11)	O16—N15—C7	118.34 (12)
C7—C8—H8	121.1	N2—O12—H12	109.5

### Hydrogen-bond geometry (Å, °)

**Table:**

*D*—H···*A*	*D*—H	H···*A*	*D*···*A*	*D*—H···*A*
O12—H12···O11^i^	0.82	1.99	2.7013 (14)	144
O12—H12···O11	0.82	2.20	2.6305 (15)	113

Symmetry codes: (i) −*x*+1, −*y*+1, −*z*+1.

## References

[bb1] Bohé, L. & Kammoun, M. (2004). *Tetrahedron Lett.* **45**, 747–751.

[bb2] Bruker (1998). *SMART and *SAINT** Bruker AXS Inc., Madison, Wisconsin, USA.

[bb3] Burnett, M. N. & Johnson, C. K. (1996). *ORTEPIII.* Report ORNL-6895. Oak Ridge National Laboratory, Oak Ridge, Tennessee, USA.

[bb4] Coppens, P., Leiserowitz, L. & Rabinovich, D. (1965). *Acta Cryst.* **18**, 1035–1038.

[bb5] Cremer, D. & Pople, J. A. (1975). *J. Am. Chem. Soc* **97**, 1354–1358.

[bb6] Farrugia, L. J. (1997). *J. Appl. Cryst.* **30**, 565.

[bb7] Farrugia, L. J. (1999). *J. Appl. Cryst.* **32**, 837–838.

[bb8] Kurzak, B., Kozlowski, H. & Farkas, E. (1992). *Coord. Chem. Rev.* **114**, 169–171.

[bb9] Miller, M. (1989). *J. Chem. Rev.* **89**, 1563–1662.

[bb10] Porcheddu, A. & Giacomelli, G. (2006). *J. Org. Chem.* **71**, 7057–7059. 10.1021/jo061018g 16930063

[bb11] Sheldrick, G. M. (2008). *Acta Cryst.* A**64**, 112–122. 10.1107/S0108767307043930 18156677

[bb12] Spek, A. L. (2003). *J. Appl. Cryst.* **36**, 7–13.

[bb13] Weber, G. (1983). *Cancer Res.* **43**, 3466–3470. 6305486

